# Supplementary material for: Understanding the synergistic effect of physicochemical properties of nanoparticles and their cellular entry pathways
Source: Commun Biol. 2020 Apr 30;3:205. doi: 10.1038/s42003-020-0917-1 (PMC7192949; doi:10.1038/s42003-020-0917-1)
Supplement: Supplementary file 1 — Supplementary Information [file 42003_2020_917_MOESM1_ESM.pdf]

Supplementary Information

Understanding the Synergistic Effect of Physicochemical Properties of Nanoparticles and their Cellular Entry Pathways

Supplementary Figures

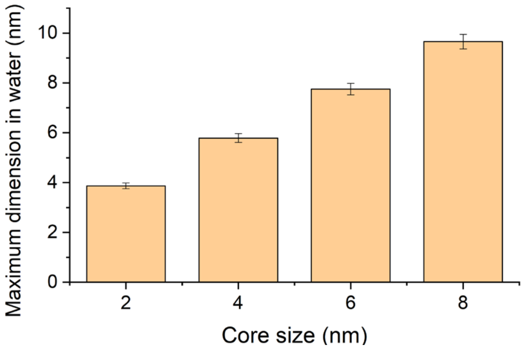

Supplementary Figure 1. Dimensions of nanoparticle in water vs. its core size. Error bars are standard deviation over 20 ns simulations.

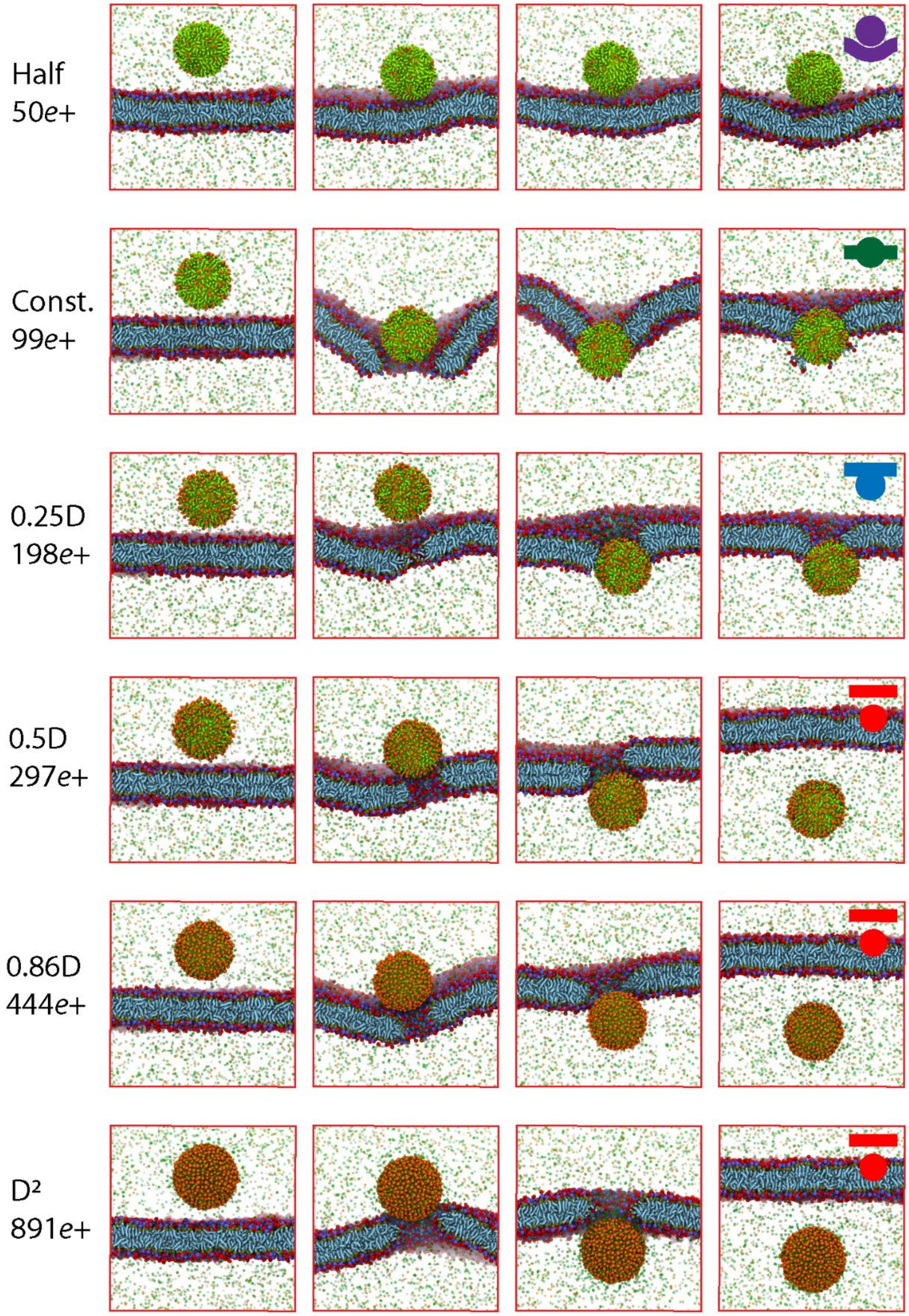

Supplementary Figure 2. Effect of nanoparticle charge/pKa on translocation. (a) Snapshots of the translocation process of nanoparticles (6 nm core and hydrophobic ligand) with varying charges across cell membrane for cellular entry.

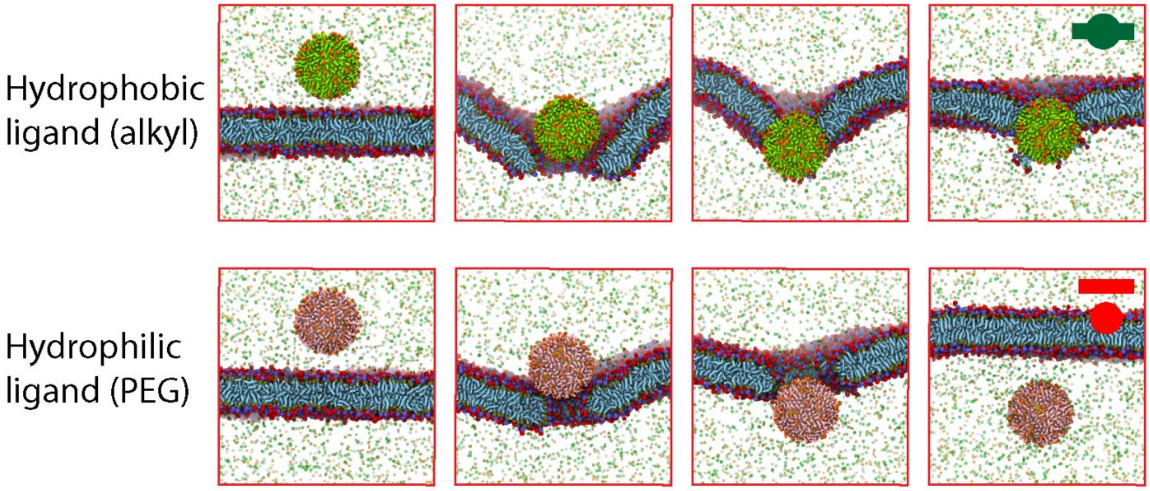

Supplementary Figure 3. Effect of nanoparticle ligand hydrophobicity on translocation. (a) Snapshots of the translocation process of nanoparticles (6 nm core and 100*e*+) with either hydrophobic ligand or hydrophilic ligand across cell membrane for cellular entry.

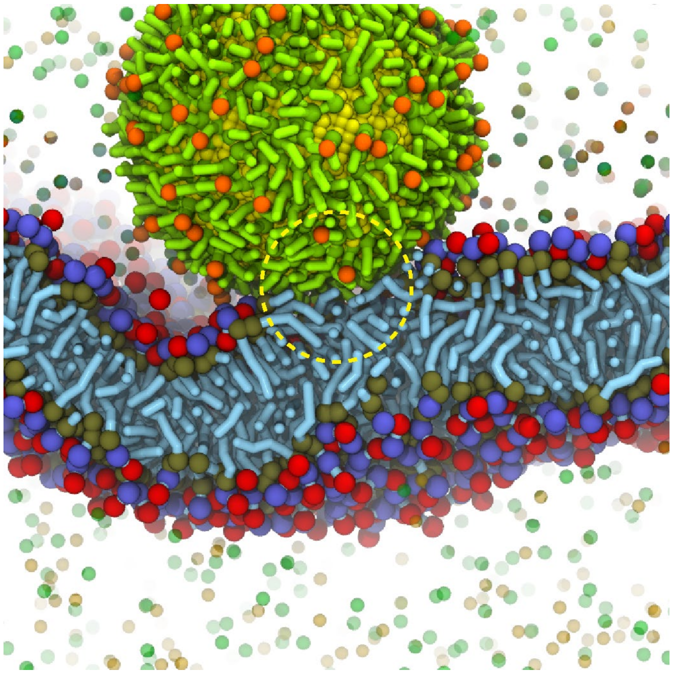

Supplementary Figure 4. Insertion of hydrophobic ligands into the non-polar interior of lipid membranes (yellow circle) before pore nucleation and subsequent translocation. (8 nm core, 100*e*+, hydrophobic ligands)

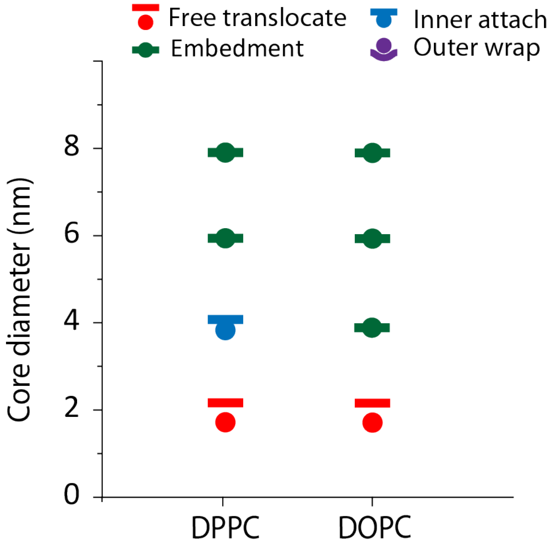

Supplementary Figure 5. Traslocation type of hydrophobic nanoparticles (from 2 nm to 8 nm) carrying 100 e surface charge with DPPC and DOPC membranes respectively.

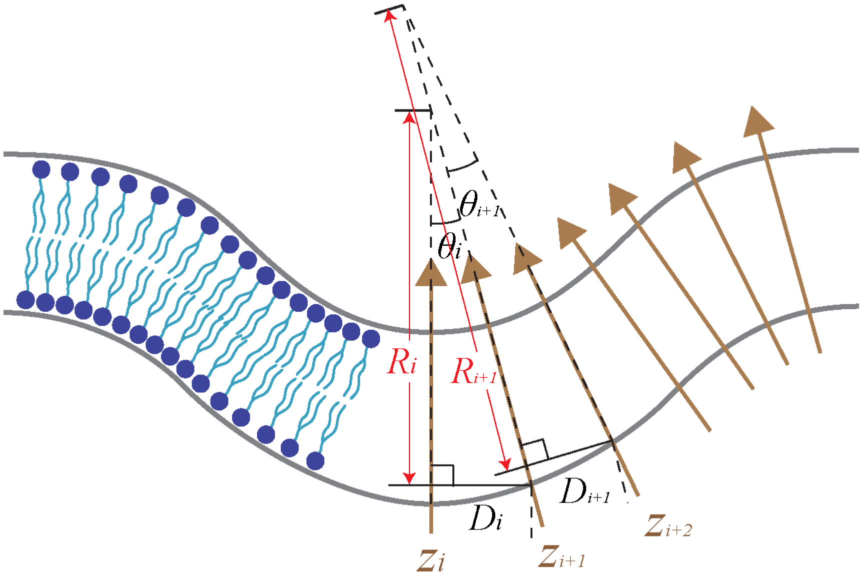

Supplementary Figure 6. Discretization method for calculation of membrane curvature. Reference normal, $\mathbf{Z_i}$, which is perpendicular to the membrane surface is calculated first to help determine θ_i and D_i. Radius of the curvature R_i is then calculated based on θ_i and D_i.

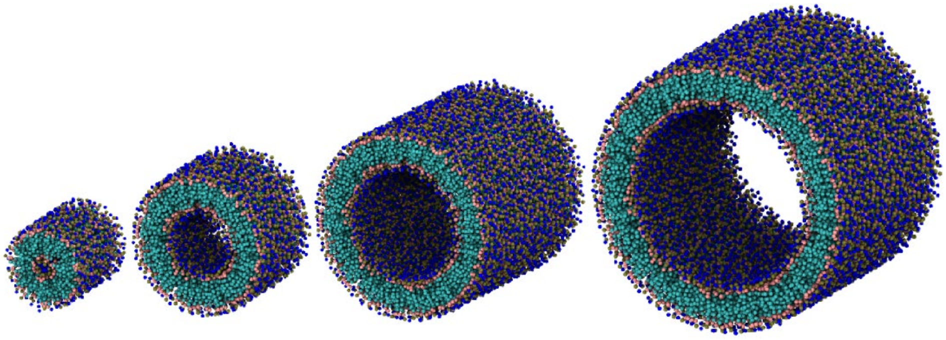

Supplementary Figure 7. Cylinder membranes with a radius of 2.5 nm, 5 nm, 7.5 nm, and 10 nm were generated using BUMPY[1] for potential of mean force calculations.

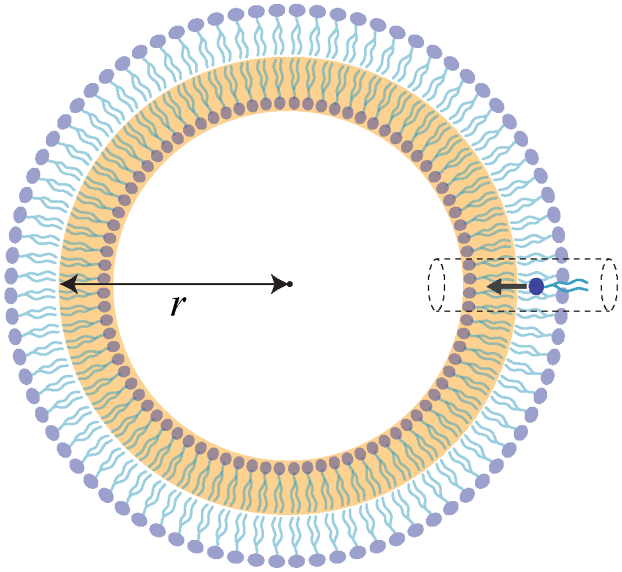

Supplementary Figure 8. Calculating PMF of lipid flip-flop on cylindrical membranes. A lipid is pulled from the outer leaflet towards the center of the cylinder that has a radius of *r* (in one dimension). The sampling interval along the pulling coordinate is 0.1 nm. The inner leaflet is position restrained by a harmonic potential with a force constant of 1000 kJ mol^{-1} nm^{-2} to prevent the membrane cylinder from deforming during the pulling. The pulled lipid is constrained to a narrow cylindrical region to eliminate lateral diffusion on the membrane during the pulling.

Supplementary Tables

Ionized ligand/total ligand	**2nm**	**4nm**	**6nm**	**8nm**
**Const.**	99/99	98/276	98/444	98/601
**Half**	50/99	50/276	50/444	50/601
**0.25 R**	99/99	149/276	198/444	248/601
**0.5 R**	99/99	198/276	297/444	396/601
**0.86 R**	99/99	276/276	444/444	601/601
**R^2**	99/99	352/352	783/783	-

Supplementary Table 1. The number of ionized ligands vs. total ligands carried by the nanoparticles with varying size and surface charge/pKa. Ligand coverage of nanoparticles (the number of ligands per surface area) decreases as the size of nanoparticles increases due to steric interactions.

**Radius (nm)**	**Curvature (nm^{-1})**	**Pivotal plane z_0 (nm)**	**Outer to inner lipid ratio**	**Total lipid**	**Outer leaflet**	**Inner leaflet**
2.50	0.40	1.00 ± 0.012	2.33	498	349	149
5.00	0.20	1.00 ± 0.012	1.50	994	596	398
7.50	0.13	1.00 ± 0.012	1.31	1494	847	647
10.0	0.10	1.00 ± 0.012	1.22	1995	1097	897
∞	0.00	/	1	128	64	64

Supplementary Table 2. Properties of membrane cylinders with different curvature generated by BUMPY.[1] The radius of cylinders and the pivotal plane of DPPC monolayer z_0 are used to determine the outer to inner leaflet lipid number ratio.
